# A TRiP RNAi screen to identify molecules necessary for *Drosophila* photoreceptor differentiation

**DOI:** 10.1093/g3journal/jkac257

**Published:** 2022-10-11

**Authors:** Johnathan Rylee, Simpla Mahato, John Aldrich, Emma Bergh, Brandon Sizemore, Lauren E Feder, Shaun Grega, Kennedy Helms, Megan Maar, Steven G Britt, Andrew C Zelhof

**Affiliations:** Department of Biology, Indiana University, Bloomington, IN 47405, USA; Department of Biology, Indiana University, Bloomington, IN 47405, USA; Department of Neurology and Ophthalmology, Dell Medical School, University of Texas, Austin, TX 78712, USA; Department of Biology, Indiana University, Bloomington, IN 47405, USA; Department of Biology, Indiana University, Bloomington, IN 47405, USA; Department of Biology, Indiana University, Bloomington, IN 47405, USA; Department of Biology, Indiana University, Bloomington, IN 47405, USA; Department of Biology, Indiana University, Bloomington, IN 47405, USA; Department of Biology, Indiana University, Bloomington, IN 47405, USA; Department of Neurology and Ophthalmology, Dell Medical School, University of Texas, Austin, TX 78712, USA; Department of Biology, Indiana University, Bloomington, IN 47405, USA

**Keywords:** *Drosophila*, differentiation, photoreceptor, RNAi

## Abstract

*Drosophila* rhabdomeric terminal photoreceptor differentiation is an extended process taking several days to complete. Following ommatidial patterning by the morphogenetic furrow, photoreceptors are sequentially recruited and specified, and terminal differentiation begins. Key events of terminal differentiation include the establishment of apical and basolateral domains, rhabdomere and stalk formation, inter-rhabdomeral space formation, and expression of phototransduction machinery. While many key regulators of these processes have been identified, the complete network of transcription factors to downstream effector molecules necessary for regulating each of these major events remains incomplete. Here, we report an RNAi screen to identify additional molecules and cellular pathways required for photoreceptor terminal differentiation. First, we tested several eye-specific GAL4 drivers for correct spatial and temporal specificity and identified Pph13-GAL4 as the most appropriate GAL4 line for our screen. We screened lines available through the Transgenic RNAi Project and isolated lines that when combined with Pph13-GAL4 resulted in the loss of the deep pseudopupil, as a readout for abnormal differentiation. In the end, we screened 6,189 lines, representing 3,971 genes, and have identified 64 genes, illuminating potential new regulatory molecules and cellular pathways for the differentiation and organization of *Drosophila* rhabdomeric photoreceptors.

## Introduction

*Drosophila* retinas are organized with ∼800 ommatidia, each containing a cluster of eight photoreceptors, and their associated retinal accessory cells, pigment, and cone cells, in a stereotyped pattern. After the passage of the morphogenetic furrow in the third larval instar eye disc, the photoreceptors are sequentially recruited, and this is followed by terminal differentiation which occurs throughout pupariation. The determination of retinal tissue and initial recruitment and specification of the eight photoreceptors is well defined [reviewed in Kumar (2010) and Chen and Desplan (2020)]. The core retinal determination gene network (RDGN) includes the transcription factors *eyeless* (ey), *twin of eyeless* (toy), *eyes absent* (eya), *dachshund* (dac), and *sine oculis* (so), and the activities of this gene network are responsible for defining retinal tissue (Halder *et al.* 1995; Chen *et al.* 1997; Halder *et al.* 1998; Czerny *et al.* 1999; Niimi *et al.* 1999). Individual photoreceptors are specified after the passing of the morphogenetic furrow (MF), initiated by the specification of the R8 photoreceptor followed by subsequent recruitment of R2 and R5, R3 and R4, R1 and R6, and finally, R7 (Tomlinson and Ready 1987).

It takes ∼2 days for the MF to pass through the eye imaginal disc and for all ommatidia to be patterned (Heberlein *et al.* 1993). By the end of the third larval instar, terminal differentiation begins and occurs throughout pupariation. Through terminal differentiation events, the photoreceptor cells obtain their distinct morphology and are properly arrayed within each ommatidium (Longley and Ready 1995). These events include the establishment of apical, stalk, and baso-lateral membranes, rhabdomere morphogenesis, apical secretion of matrix material to form the IRS, synapse formation, and expression and delivery of the phototransduction machinery to the rhabdomere [reviewed by Chen and Desplan (2020)]. An important molecular switch for initiating terminal differentiation is the activation of the eye-specific transcription factor *glass* by the RDGN cofactors *sine oculis* and *eya* (Bernardo-Garcia *et al.* 2016). Glass serves 2 separable roles in the proper development of the retina. First, it is required for patterning the different cell types within ommatidia after the passage of the morphogenetic furrow (Bernardo-Garcia *et al.* 2016; Liang *et al.* 2016; Morrison *et al.* 2018). Both the number and distribution of both neuronal and non-neuronal cell types within ommatidia are altered in *glass* mutants (Bernardo-Garcia *et al.* 2016; Liang *et al.* 2016; Morrison *et al.* 2018). Next, it activates the genetic program to initiate terminal differentiation. Although identifiable neuronal cells persist within *glass* mutant ommatidia into pupariation, they fail to obtain terminal photoreceptor morphological features, and cell type identity (Bernardo-Garcia *et al.* 2016; Liang *et al.* 2016).

Besides Glass, terminal differentiation is also coordinated by 2 homeodomain proteins, Orthodenticle (Otd) and Pph13; *Pph13* is a direct transcription target of Glass (Bernardo-Garcia *et al.* 2016; Liang *et al.* 2016). Pph13 and Otd are key regulators of the metamorphosis of the apical membrane into rhabdomeres (Vandendries *et al.* 1996; Tahayato *et al.* 2003; Zelhof *et al.* 2003; Mishra *et al.* 2010), and for directing the expression of phototransduction machinery (Tahayato *et al.* 2003; Mishra *et al.* 2010). Many transcriptional targets of Glass, Pph13, and Otd have been identified (Naval-Sánchez *et al.* 2013; Mencarelli and Pichaud 2015; Liang *et al.* 2016) and are involved in a variety of processes of photoreceptor terminal differentiation, including rhabdomere formation (Chaoptin, Prominin), phototransduction (opsins, Arr1, InaD), and division/differentiation of the apical membrane (PIP82). In essence, transcriptional profiling of these transcription factors has defined many photoreceptor-specific genes required for photoreceptor function and morphology.

However, absent from these data sets are genes that are not photoreceptor specific but are, nevertheless, critical for both the morphology and organization of photoreceptors in each ommatidium. For example, genes involved in trafficking, secretion, and establishing apical/basal polarity are entwined with photoreceptor terminal differentiation while also being critical for many fundamental cellular processes in many different tissues. Rhabdomeric photoreceptors have distinct membrane domains, including the basolateral and apical membranes, which are further segregated into the rhabdomeric and stalk portions. The apical and basolateral membranes are separated by the zonula adherens (ZA) which become localized to separate the two domains during terminal differentiation by the activities of the Par complex and associated proteins [reviewed by Pichaud (2018)]. Crumbs are necessary for sequestering the Cdc42-Par6-aPKC-Baz complex to the apical surface. aPKC phosphorylation of Baz causes its exclusion from the apical domain, and it becomes localized, along with Armadillo and other adherens junction proteins, to the ZA (Walther and Pichaud 2010). Crumbs become localized to the stalk where it is essential for proper stalk membrane formation and maintenance (Pellikka *et al.* 2002) where the recruitment of aPKC limits the spread of rhabdomeric targeted proteins, e.g. PIP82 (Zelhof *et al.* 2020). The distinct photoreceptor membrane domains also house unique proteins thus requiring a sorting system necessary for both proper morphology and function of photoreceptors. To date, many genes have been identified which are involved in apical trafficking and/or secretion, or in the transport and assembly of the microvilli (Zelhof and Hardy 2004; Iwanami *et al.* 2016; Laffafian and Tepass 2019). For example, the apical transport of rhodopsin occurs via vesicles trafficked along the fibers of the rhabdomere terminal web (RTW) (Chang and Ready 2000) requiring the activity of several proteins including MyoV, Rab11, and dRip11 (Li *et al.* 2007).

Despite the characterization of many genes required for the various aspects of photoreceptor terminal differentiation, there remains a gap in our understanding of the molecules responsible for temporal control of terminal differentiation events, the suite of proteins necessary for trafficking and secretion, and the coordination between the two. To address this gap, we conducted an RNAi screen to identify transcriptional regulators, and components of trafficking and secretory pathways necessary for proper photoreceptor morphogenesis and organization but not synaptic connectivity. We used Pph13-GAL4 to drive 6,189 TRiP RNAi lines targeting 3,971 genes for a knockdown in developing photoreceptors during the process of terminal differentiation. Given, that several cellular processes are required for proper photoreceptor morphogenesis, we utilized the appearance of the deep pseudopupil (DPP) as a catch-all readout for proper organization and morphology of the photoreceptors. We identified 64 genes that resulted in a change in the appearance of the DPP. Subsequent sampling of a subset with transmission electron microscopy (TEM) confirmed and revealed the defects in terminal differentiation identifying additional molecules and cellular processes required for rhabdomeric photoreceptor development.

## Materials and methods

### Drosophila strains and husbandry

The following stocks were utilized in this study: Pph13-GAL4 (Mahato *et al.* 2014), GMR-GAL4 Long (RRID: BDSC 8605) and GMR-GAL4 Short (RRID: BDSC 1104), Chp-GAL4 (PMID: 24385925), 10XUAS-IVS-GFP (RRID: BDSC 32201), EYS RNAi. (RRID: BDSC 33766), UAS-EcR-DN (RRID: BDSC 6469, RRID: BDSC 6470), *sevenless*^21^ (RRID: BDSC 40932). All TRiP lines screened are listed in Supplementary Tables 1 and 2 and all were obtained from the Bloomington Drosophila Stock Center. For RNAi expression in photoreceptors, we crossed homozygous females w*;+/+; Pph13-GAL4/Pph-13-GAL4 to males from each RNAi line. All crosses were maintained at 25°C and on a 12-h light/dark cycle.

### Testing GAL4 lines

Females of GMR-GAL4, long and short, Pph13-GAL4, and Chp-GAL4 were crossed to male 10XUAS-IVS-GFP flies. Eye discs were collected from third instar larvae, and pupal retinas were collected at 48 and 72 h after puparium formation (APF) and processed as described in Nie *et al.* (2015) and imaged for GFP and ELAV expression.

### Deep pseudopupil detection

For each RNAi cross, at least 3 adult female progeny heads (*w*+ retinas) were scored for the misconfiguration, absence, or presence of the deep pseudopupil within 1–3 days of posteclosion. The heads were positioned on a microscope slide and examined under 10X magnification (Zeiss Plan-ACHROMAT 10×/0.25 lens) on a Zeiss Primo Star Binocular Microscope with white light illumination. If the 3 heads lacked a normal deep pseudopupil, the RNAi line was scored as a positive hit. A subset of positive hit retinas of adult female progeny were examined by TEM to reveal and confirm any defects.

### TEM and immunofluorescence staining

All procedures (TEM and Immunofluorescence) were performed as previously described (Nie *et al.* 2012, 2014). For TEM, heads taken from flies 1–2 days of posteclosion. The antibodies used in this study were: rat anti-Elav (7E8A10, 1:100, Developmental Studies Hybridoma Bank) primary antibody, mouse anti-EYS (21A6, 1:50, Developmental Studies Hybridoma Bank), rabbit anti-Rh6 (1:1,000, Dr. Claude Desplan), mouse anti-Rh5 (1:50, Dr. Steve Britt), Cy5 conjugated donkey antirat secondary (1:200, Jackson Immunoresearch #712-175-153), Alexa 488 conjugated goat antirabbit (1:200, Life Technologies #A-11008), Alexa 488 conjugated goat antimouse (1:200, Jackson Immunoresearch #115-545-146), and Cy5 conjugated goat antimouse (1:200, Jackson Immunoresearch #115-175-166). F-actin was labeled using rhodamine-conjugated phalloidin (1:200, Life Technologies #R415). Confocal images were captured on a Leica TCS SP5. TEM imaging was conducted with a JOEL 1010 and JOEL 1400. All images were processed in Fiji or Adobe Photoshop.

### Generation of mamo mutant lines

Mutations were introduced to *mamo* by crossing a Weizmann Knockout Project line (Meltzer *et al.* 2019) ubiquitously expressing gRNAs to *mamo* (RRID: BDSC 84157) with a line expressing Cas9 in germline cells from the *nanos* promoter (RRID: BDSC 54591). Female progeny were then crossed to an FM7c balancer line (RRID: BDSC 3378). Individual female progeny of this cross-containing potential mutations over the balancer chromosome were crossed to FM7c males. All crosses that yielded only FM7c males contained potential lethal *mamo* mutations. Genomic DNA was extracted from 3 female progeny of each of these crosses by crushing them in DNA extraction buffer (100 mM Tris-HCl, 50 mM EDTA, 1% SDS) heating at 95°C for 5 min, then incubating at 37° with proteinase K for 1 hour. Proteinase K was inactivated by heating at 95°C for 5 min. The extracted gDNA was then purified using the Genomic DNA Clean and Concentrator kit (Zymo Research) following the manufacturer’s instructions. Sequences spanning the gRNA target sites were PCR amplified, and Sanger sequenced using the primers listed in Supplementary Table 3. Mutations were identified and characterized using Sequencher (Gene Codes Corp.), and alignments were made using SnapGene (Dotmatics). *mamo^7^* was recombined onto FRT19A chromosome (RRID: BDSC 1709). To generate mosaic retina clones w*, *mamo*^7^ FRT 19A was crossed to P{ry[+t7.2]=ey-FLP.N}2, w[*], P{w[+mC]=tubP-GAL80}LL1P{ry[+t7.2]=neoFRT}19A (RRID: BDSC 42717).

### Quantification of opsin expression

Ten to 20 eyes were dissected from ∼2- to 5-day-old females. Ommatidia were dissociated as previously described (Chou *et al.* 1996). Opsins were detected in various combinations using fluorophore-conjugated monoclonal mouse antibodies (diluted 1:100) as previously described (Feiler *et al.* 1992; Chou *et al.* 1999; Earl and Britt 2006). In some cases, Rh4 was detected indirectly using Rb anti-Rh4 (1:500) and Goat anti-Rb-alexa 647 (1:1,000; Invitrogen).

Images were acquired via tile scan on the Nikon Ni-E FLM and quantified manually in ImageJ with help from the “Cell Counter” feature. Dorsal rim ommatidia (DRA) were ignored and R7s coexpressing Rh3 and Rh4 were counted as Rh4 expressing for statistical purposes (Mazzoni *et al.* 2008). The statistical analysis was performed in R via RStudio. dplyr was utilized to process the data, Hmisc to calculate summary stats (proportion and 95% CI), and prop.test (from the stats package) to calculate *P*-values.

## Results and discussion

### RNAi screen to identify transcriptional regulators of photoreceptor differentiation

In order to identify additional proteins necessary for the transcriptional control of photoreceptor differentiation and effector molecules for photoreceptor morphogenesis and organization, a loss-of-function genetic approach that would bypass potential roles in photoreceptor specification as well as potential pleiotropic effects in other tissues was required. Although previous mosaic screens (Xu and Rubin 1993; Germani *et al.* 2018) alleviated pleiotropic concerns by targeting the loss-of-function mutations to a particular tissue or particular cells, they did not discriminate potential temporal functions between specification and differentiation nor did they distinguish between effects specific to photoreceptors vs the accessory cells of the retina (e.g. cone or pigment cells). As such, our approach needed to combine the ability to create a loss-of-function phenotypes with specific spatial and temporal resolution. To accomplish this, we chose to perform an RNAi screen.

**Fig. 1.**
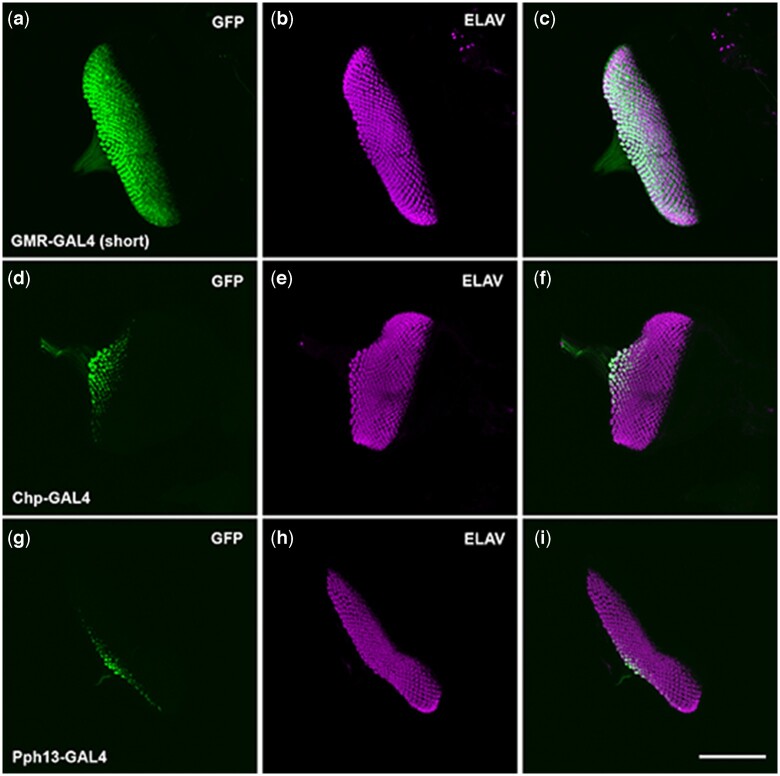
GFP reporter expression of retinal-specific GAL4 lines in the third instar eye imaginal disc. a–c) GMR-GAL4 (short) driving GFP expression, green, counterstained with Elav, magenta. d–f) Chp-GAL4 driving GFP expression counterstained with Elav. g–i) Pph13-GAL4 driving GFP expression countered stained with Elav. Scale bar 100 μm.

**Fig. 2.**
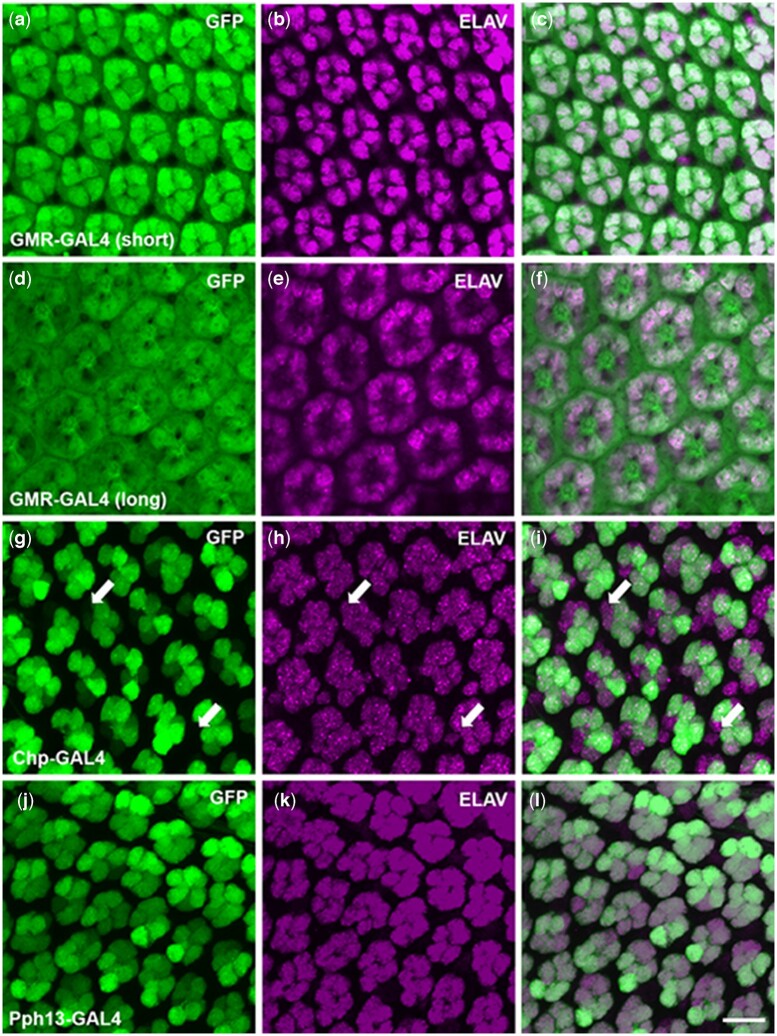
GFP reporter expression of retinal specific GAL4 lines in the developing retina at 48 h APF. a–c) GMR-GAL4 (short) driving GFP expression, green, countered stained with Elav, magenta. d–f) GMR-GAL4 (long) driving GFP expression, counterstained with Elav. g–i) Chp-GAL4 driving GFP expression counterstained with Elav. k, l) Pph13-GAL4 driving GFP expression counterstained with Elav. As a note, an occasional ELAV positive but GFP negative photoreceptor can be observed at this time point with use of Pph13-GAL4. Scale bar 10 μm.

In Drosophila, an RNAi screen is dependent upon two components. The first component is a library of RNAi resources targeting the genes of the Drosophila genome. For this, we utilized the TRiP RNAi resources (Perkins *et al.* 2015). TRiP RNAi resources are designed such that the expression of the RNAi molecules is transcriptionally regulated by GAL4 (Brand and Perrimon 1993) and, therefore, will only be expressed in cells in which GAL4 is present. In this manner, with the correct GAL4 line we can achieve the necessary temporal and spatial resolution required to generate loss-of-function phenotypes. For our purposes, the GAL4 driver should only be expressed in photoreceptors and expression should initiate around the time of differentiation, i.e. early pupal development. As previously described by Longley and Ready (1995), the late larval to early pupal transition marks the start of the critical time frame in which the retinal epithelial monolayer transforms and reorganizes to the 3D organization observed in the adult retina. To date, there are a few well-established and utilized GAL4 lines for expression in retinal tissues GMR-GAL4, long and short versions (Ellis *et al.* 1993; Wernet *et al.* 2003), Chp-GAL4 (Mishra *et al.* 2013), Pph13-GAL4 (Nie *et al.* 2012), and Rh1-GAL4 (Mollereau *et al.* 2000). GMR-GAL4 short consists of only five tandem repeats for the binding of the retinal specific transcription factor Glass (Ellis *et al.* 1993). GMR-GAL4 long contains five copies of a 38-bp sequence from the *ninaE* promoter that contains a Glass binding site (Wernet *et al.* 2003). Chp-GAL4 consists of a 4.5 kb upstream sequence of the photoreceptor-specific adhesion molecule Chaoptin. Pph13-GAL4 represents an 811 bp upstream sequence from the photoreceptor-specific homeodomain-containing protein Pph13. For our purposes, we eliminated the possibility of utilizing Rh1-GAL4 since the expression is limited to only the outer R1–R6 photoreceptors, and expression is activated well after differentiation has initiated. To determine which of the remaining GAL4 lines would best fit our criteria, we examined GAL4 expression utilizing GFP as a readout of activity. We examined the expression of GFP at several different time points: third instar larval eye imaginal discs (Fig. 1), 48 h (Fig. 2), and 72 h (Supplementary Fig. S1) APF.

During larval development, as previously described, we found GMR-GAL4 short activates reporter expression as the morphogenetic furrow passes in larval eye discs (Fig. 1, a–c) and overlaps with the initiation of ELAV expression as the photoreceptor neurons are recruited and specified. Given that both *chaoptin* and *Pph13* are downstream transcriptional targets of Glass (Liang *et al.* 2016; Morrison *et al.* 2018), we expected both to initiate expression later than GMR. As such, both Chp-GAL4 (Fig. 1, d–f) and Pph13-GAL4 (Fig. 1, g–i) activated expression later in larval eye discs, and multiple rows of patterned ommatidia could be seen between the morphogenetic furrow and ommatidia containing GFP labeled cells.

At 48 h APF, we observed notable differences between the GAL4 lines. First, GMR-GAL4, long or short, reporter expression was not limited to photoreceptors and was observed in the surrounding pigment cells (Fig. 2, a–f). Our results with either GMR-GAL4 line confirmed that neither line is limited to only photoreceptors (Escobedo *et al.* 2021) in the developing retina. Hence, the early expression of the GMR-GAL4 lines and expression outside of photoreceptors would potentially interfere with earlier patterning and specification events as well as the differentiation of other cell types in the retina. Although Chp-GAL4 was limited to photoreceptors, expression was inconsistent. At 48 h, APF we observed many photoreceptors lacking GFP expression in Chp-GAL4 retinas (Fig. 2, g–i). Only Pph13-GAL4 drove consistent reporter expression and expression was limited to photoreceptors (Fig. 2, j–l). By 72 h, APF GFP expression was detected in all photoreceptors in all 4 GAL4 lines (Supplementary Fig. 1). Based on these results, we decided to utilize Pph13-GAL4 as our driver line.

**Fig. 3.**
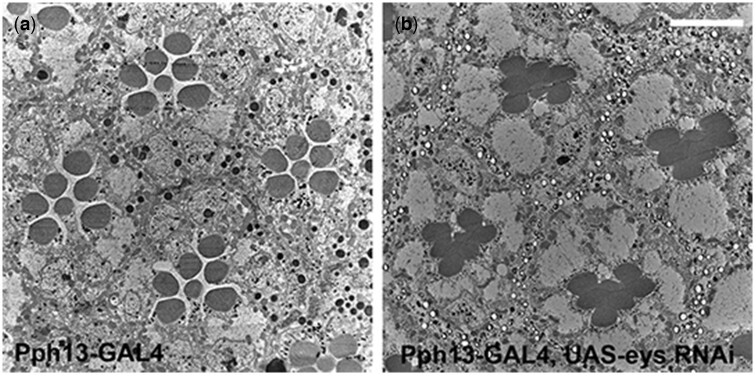
Confirmation that Pph13-GAL4 elicits an RNAi defect in differentiation. a) Transmission electron microscopy micrograph of a Pph13-GAL4 heterozygote adult retina. b) Transmission electron microscopy micrograph of a Pph13-GAL4 driving expression of *eys* RNAi. Note the absence of the inter-rhabdomeral space and the juxtaposed rhabdomeres. Scale bar 5 μm.

To demonstrate Pph13-GAL4 could effectively target terminal differentiation events, we tested whether driving *eys* RNAi would generate an *eys* mutant phenotype. We chose only to test EYS because previously we demonstrated that utilizing GMR-GAL4 to drive RNAi constructs produces phenotypes for *eys*, *chaoptin*, *sev*, *TRPgamma*, *inaC*, *arr2*, *Gqalpha*, and *Plc21c* (Ni *et al.* 2009) confirming the feasibility of a RNAi approach. EYS is a secreted extracellular matrix protein and is responsible for generating the inter-rhabdomeral space (IRS), the extracellular matrix that separates and positions the developing rhabdomeres in their stereotypical positions within each ommatidium (Husain *et al.* 2006; Zelhof *et al.* 2006). The loss of *eys* results in all of the rhabdomeres within an ommatidium remaining juxtaposed to each other (Husain *et al.* 2006; Zelhof *et al.* 2006). Moreover, EYS can be detected as early as 36 h APF and is a key marker of the morphological differentiation of photoreceptors (Nie *et al.* 2014). When Pph13-GAL4 is utilized to drive *eys* RNAi, we observed a loss-of-function *eys* phenotype (Fig. 3b), as compared to control (Fig. 3a) demonstrating Pph13-Gal4 is capable of inducing defects in photoreceptor terminal differentiation.

**Fig. 4.**
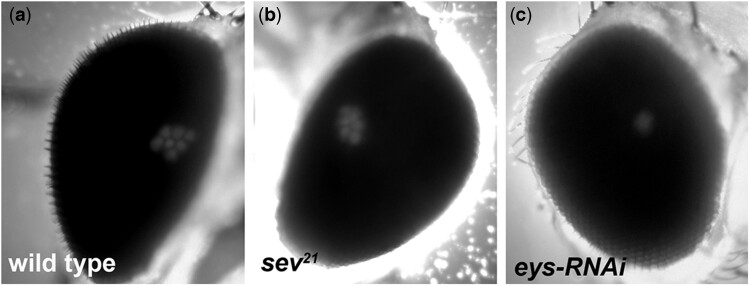
Examples of wild type and abnormal deep pseudopupils. a) Deep pseudopupil (DPP) of a wild-type pigmented retina, progeny of Pph13-GAL4 crossed to Cabton-S. Note the distinct trapezoidal arrangement of 6 outer light spots and 1 inner light spot. b) Deep pseudopupil of a *sevenless^21^* mutant retina. The loss of the R7 photoreceptor results in abnormal deep pseudopupil with only 6 distinct light spots. c) Deep pseudopupil of Pph13-GAL4 driving expression of *eys* RNAi. The deep pseudopupil is reduced to a single light spot.

Whereas TEM provides a very accurate view of the potential morphological condition and arrangement of photoreceptors and associated rhabdomeres, to perform a screen with TEM as a phenotypic readout would be temporally inefficient and cost prohibitive. Immunofluorescence of adult retinas also provides an accurate view of the cellular organization and development of the photoreceptors but, like TEM would not be an efficient method for phenotypic readout. Given that our screen has the potential to affect multiple cellular pathways that would lead to changes in photoreceptor architecture and organization (Longley and Ready 1995), i.e. rhabdomere morphology, rhabdomere position, defects in IRS formation, and photoreceptor cell polarity, we needed a general catch-all scorable phenotype. We chose to score for the absence or presence of the deep pseudopupil (DPP) in 1- to 2-day-old adults (Franceschini and Kirschfeld 1971; Franceschini 1972) (Fig. 4a); the visualization of the DPP is an efficient and sensitive method to assess the organization and morphology of the photoreceptors. To test if the DPP is an appropriate phenotypic readout, we compared the DPP formed in wild type, *sevenless* mutants, and in retinas expressing *eys* RNAi. In both, the *sevenless* mutant (Fig. 4a) and *eys* RNAi retinas (Fig. 4c), we can clearly observe a change in the DPP. In *sevenless* mutant, only 6 distinct spots are detected as expected (Tomlinson and Ready 1986), and in *eys* RNAi the DPP has been reduced to one central large spot. Therefore, as a readout for our screen, we screened for RNAi lines that disrupted the formation of the DPP causing it to be either absent or fuzzy. Moreover, we did not expect to observe retinas in which the overall outward appearance and integrity of the retina were disrupted. Pph13-GAL4 is expressed after the photoreceptors are specified and is limited only to the photoreceptor cells thus avoiding any changes in the specification of photoreceptors or differentiation of the accessory cells of the retina that could result in a rough eye appearance.

### Transcription factors with RNAi phenotype

There are over 15,000 RNAi lines available from the TRiP collection. To select the RNAi lines to screen for transcriptional regulators of differentiation, we searched the DRSC/TRiP functional genomics resource site (https://fgr.hms.harvard.edu/trip-rnai-fly-stocks; accessed 2022 September 28) for RNAi lines against transcription factor/DNA binding genes identified using Gene List Annotation for Drosophila (GLAD, https://www.flyrnai.org/tools/glad/web/; accessed 2022 September 28). We identified 1,841 RNAi lines targeting 1,172 genes with predicted DNA binding activity (Supplementary Table 1). Of these lines, eight resulted in a misconfigured DPP phenotype as described below. For each of these eight genes, only one RNAi line for each resulted in a photoreceptor phenotype. A summary of each hit and the knockdown phenotype follows.

#### Cdc5

**Fig. 5.**
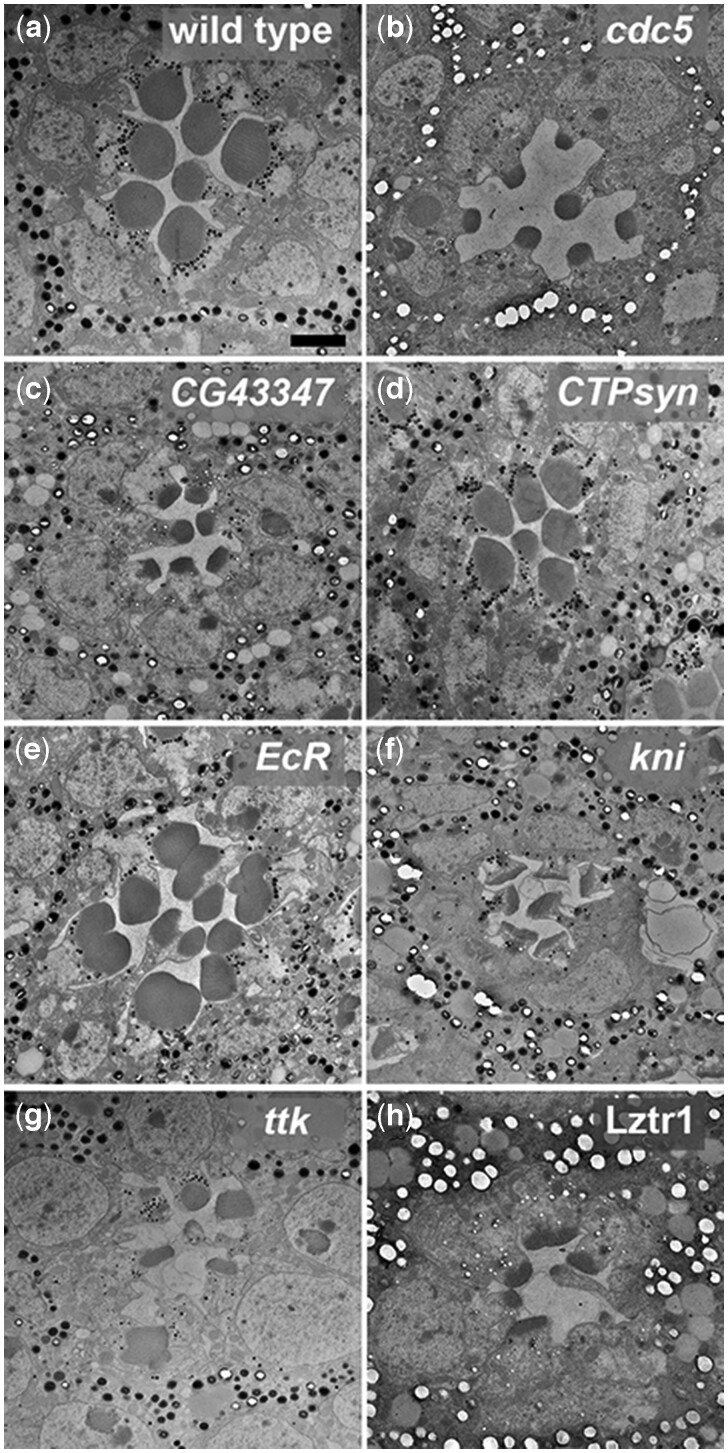
Transmission electron microscopy analysis of RNAi transcription factor phenotypes. Photoreceptor and rhabdomere structure in a single ommatidium of wildtype (Pph13-GAL4 heterozygote) (a), RNAi *cdc5* (b), RNAi *CG43347* (c), RNAi *CTPsyn* (d), RNAi *EcR* (e), RNAi *knirps* (kni) (f), RNAi *tramtrack* (ttk) (g), and RNAi *Lztr1* (h). Scale bar 2 μm.

Cdc5 is a member of the Prp19 complex which is involved in a variety of activities including mRNA splicing, lipid droplet biosynthesis, and DNA repair (Mount and Salz 2000; Chanarat and Sträßer 2013). This gene is homologous to human CDC5L and S. pombe Cdc5, not the *Saccharomyces cerevisiae* Cdc5 Polo kinase. *Cdc5* RNAi photoreceptors had reduced rhabdomeres (Fig. 5b) as compared to the control (Fig. 5a).

#### CG43347

CG43347 is a zinc finger transcription factor. The regulatory targets of CG43347 are unknown, and it has no previously reported associated phenotypes. Like Cdc5, CG43347 knockdown resulted in severely reduced rhabdomere size (Fig. 5c).

#### CTPsyn

CTPsyn does not have any predicted activity as a transcription factor. It was identified as a transcription factor by the GLAD database because it contains a bHLH domain. While this domain is associated with DNA binding, it also facilitates protein-protein interactions. CTP synthase is involved in *myc*-mediated control of cell size (Aughey *et al.* 2016), and regulation of CTPsyn by miR-975 controls cell proliferation and differentiation (Woo *et al.* 2019). While we observed no cell size or proliferation defects, CTP synthase knockdown resulted in aberrant rhabdomere morphology. Qualitatively, rhabdomeres had a more rectangular shape than the round shape seen in wild-type flies (Fig. 5d).

#### EcR

EcR is the primary receptor for the hormone ecdysone. It is known to work in concert with Otd to repress Kr-H1 expression during terminal photoreceptor differentiation to allow photoreceptor maturation to proceed (Fichelson *et al.* 2012). Diverse roles in neuronal development and fate selection including that of photoreceptors have been demonstrated (Schubiger *et al.* 1998; Schubiger *et al.* 2005; Sprecher and Desplan 2008). Photoreceptors in EcR knockdown flies showed a severe apical membrane defect with multiple rhabdomeres forming on individual photoreceptors (Fig. 5e). To determine if this unique phenotype was due to off-target effects, we asked whether the maturation defects that we see in *EcR*-RNAi photoreceptors is phenocopied by driving expression of EcR dominant negative alleles (EcR-DN) using Pph13-GAL4. We found a similar apical domain defect associated with both *EcR*-RNAi and EcR-DN expression (Supplementary Fig. 2a).

#### Knirps

Knirps is a short-range transcriptional repressor. It is one of the gap repressors that are key to abdominal segment development (Nauber *et al.* 1988; Pankratz *et al.* 1989), and it plays a role in the development of many structures including, but not limited to the head (Gonzalez-Gaitan *et al.* 1994), wing veins (Lunde *et al.* 1998; Lunde *et al.* 2003), and trachea (Chen *et al.* 1998). In *knirps* knockdown photoreceptors, the rhabdomeres have elongated/bulbous submicrovillar cisternae and severely shortened and poorly organized rhabdomere microvilli (Fig. 5f and Supplementary Fig. 2b). Additionally, we observed vacuoles or pools at the bases of some photoreceptors.

#### Tramtrack

Tramtrak (ttk) has long and short isoforms called ttk88 and ttk69, respectively (Xiong and Montell 1993). Ttk88 suppresses R7 cell fate, and loss of Ttk88 results in ectopic R7 cells. Ttk69 is highly expressed in all photoreceptors during pupariation throughout terminal differentiation (Lai and Li 1999). Mutant clones which remove both isoforms fail to differentiate, and the photoreceptors degenerate rapidly (Xiong and Montell 1993). The *ttk* RNAi line that yielded a DPP-phenotype in our screen targets a common exon present in both transcripts. Knockdown of *ttk* using this line results in aberrant rhabdomere formation. The apical membranes seem to be poorly specified resulting in malformed and misplaced rhabdomeres within ommatidia (Fig. 5g). The supernumerary R7 phenotype associated with loss of Ttk88 was not obvious due to the general disruption in differentiation. We suspect that the knockdown phenotype we observed represents a necessary role that Ttk69 plays in terminal differentiation. Ttk69 has a known role in epithelial tube formation, and one report identified LAMA, Shi, and Dynamin as downstream effectors (Peters *et al.* 2013). These results and our result suggest that Ttk69 could be a key regulator of apical trafficking.

#### Lztr1

Lztr1 is a leucine zipper-like transcription factor that is a negative regulator of Ras signaling. Lztr1 regulates Ras through ubiquitination (Bigenzahn *et al.* 2018; Abe *et al.* 2020) and Drosophila Lztr1 mutants show an ectopic wing vein Ras gain of function phenotype (Bigenzahn *et al.* 2018). Additionally, Lztr1 was recently identified as a regulator of circadian rhythms affecting sleep and metabolism (Maurer *et al.* 2020). RNAi knockdown of Lztr1 in photoreceptors resulted in smaller malformed rhabdomeres (Fig. 5h).

#### Mamo

*Drosophila mamo* encodes a zinc finger BTB transcription factor that is required for meiosis (Mukai *et al.* 2007). Additionally, *mamo* is a key factor in the fate selection of mushroom body neurons and neuronal remodeling (Alyagor *et al.* 2018; Liu *et al.* 2019). The striking phenotype we observed was a severe disruption of polarity resulting in aberrant rhabdomere formation, placement, and fusion, and EYS secretion is not limited to the apical domain (Supplementary Fig. 3, a and b). In addition, we observed a loss of Rh6 opsin expression suggesting a potential defect in R8 subtype specification. The *Drosophila* retina predominantly consists of two ommatidial subtypes which can be distinguished based on the combination of opsins expressed in the R7 and R8 photoreceptors. Approximately 70% are classified as “Yellow” and express Rh4 in R7 paired with Rh6 in R8. The other 30% are “Pale” ommatidia, which express Rh3 in R7 and Rh5 in R8 (Franceschini *et al.* 1981; Chou *et al.* 1996). Subtype specification occurs stochastically in the R7 and this info is conveyed to the R8 via an inductive signal resulting in paired opsin expression (Papatsenko *et al.* 1997; Chou *et al.* 1999; Wernet and Desplan 2004). Thus, in wild-type retinas, the R8 photoreceptor either expresses Rh6 or Rh5 opsin resulting in a mosaic pattern in the eye (Supplementary Fig. 3, c and d). However, in the putative knockdown of *mamo*, Rh6 expression was lost and only Rh5 expression was detected (Supplementary Fig. 3, e–g). We examined this further by immunostaining opsins in dissociated ommatidia (Supplementary Fig. 4a). RNAi knockdown of *mamo* resulted in exclusive (100%) Rh5 expression in R8 photoreceptors. In contrast, we observed a relatively small but significant increase in the proportion Rh3 vs Rh4 expressing R7 photoreceptors in *mamo*-RNAi flies vs controls (51% vs 43% Rh3; *P* = 0.016) (Supplementary Table 4). Furthermore, in *mamo*-RNAi flies, we observed a dramatic increase in Rh4/Rh5 mispairing events vs controls (Supplementary Fig. 4b; 48.6% vs 0.2%), which demonstrates that the increase in Rh5-expressing R8s is due to a decoupling of cell fate decisions occurring in the R7 and R8 cells resulting in the misexpression of Rh5 and repression of Rh6 in Yellow ommatidia. The low level (∼10%) of Rh3/Rh6 mispairing that is typically seen in most genotypes, including our control, is absent in the mamo-RNAi flies.

To determine if this dramatic phenotype is the result of the loss of *mamo* function, we generated several *mutant* lines of *mamo* by crossing an available guide RNA line from the Weizmann Knockout Project (Meltzer *et al.* 2019) to a line expressing Cas9 in germline cells, since the original *mamo* alleles (Alyagor *et al.* 2018) no longer exist. We recovered three mutant alleles (Supplementary Fig. 5a), and allele “seven” was recombined onto an FRT chromosome to determine the role of *mamo* in photoreceptors. Mosaic analysis determined that the aberrant defects in polarity were not found in mutant photoreceptors (Supplementary Fig. 3h). Also, we did not observe any change in opsin expression (data not shown). We also assessed phenotypes in another RNAi line that has been reported to give a *mamo* phenotype (Alyagor *et al.* 2018; Liu *et al.* 2019) but did not see any change in morphology or opsin expression (data not shown). Therefore, the phenotype we observed using RNAi line 60111 is probably due to an off-target effect and not caused by *mamo* knockdown.

### Cellular trafficking, transport, secretion genes with RNAi phenotypes

A critical aspect of photoreceptor differentiation is the generation and maintenance of apical/basal polarity, the localization and turnover of proteins to distinct membrane domains, and the secretion of an extracellular matrix for the proper morphology and positioning of the photoreceptor rhabdomeres. To query genes potentially involved in these processes we generated a target list utilizing Flybase Gene Ontology (GO) terms (Supplementary Table 2) and tested the identified positive hits from four different RNAi screens in Drosophila tissue culture cells designed to reveal components of the secretory pathway, and regulation of wingless and hedgehog processing and secretion (Nybakken *et al.* 2005; Bard *et al.* 2006; Wendler *et al.* 2010; Port *et al.* 2011). Together, this generated an additional 4,348 TRiP lines to a screen representing 2,799 genes. From this collection, we identified 56 genes that when knocked down resulted in aberrant formation of the deep pseudopupil (Table 1) and can be divided into 10 broad functional groups (Supplementary Fig. 6). Genes were placed into functional groupings using gene lists available from GLAD. Remaining genes were placed into the appropriate groups using associated GO terms, and previously reported functional data. To confirm and characterize the defects associated with the loss or aberrant deep pseudopupil, we analyzed a sample by TEM. Below, we describe a selection of phenotypes associated with genes from several different functional groups.

**Table 1.** List of RNAi lines resulting in dpp phenotype.

Gene symbol	FlyBase ID	Stock ID	Functional group
alphaCOP	FBgn0025725	RRID: BDSC_34923	Autophagy
beta'COP	FBgn0025724	RRID: BDSC_36113	Autophagy
CG6512	FBgn0036702	RRID: BDSC_34343	Autophagy
Garz	FBgn0264560	RRID: BDSC_34987	Autophagy
RpL10Ab	FBgn0036213	RRID: BDSC_34695	Autophagy
Sec61beta	FBgn0010638	RRID: BDSC_50626	Autophagy
Snap29	FBgn0034913	RRID: BDSC_51893	Autophagy

Hsc70-3	FBgn0001218	RRID: BDSC_80420	Chaperone

Act5C	FBgn0000042	RRID: BDSC_42651	Cytoskeleton
Act88F	FBgn0000047	RRID: BDSC_60347	Cytoskeleton
alpha-Spec	FBgn0250789	RRID: BDSC_42801	Cytoskeleton
	FBgn0250789	RRID: BDSC_56932	Cytoskeleton

Acbp5	FBgn0035926	RRID: BDSC_63633	Metabolic
Acsl	FBgn0263120	RRID: BDSC_41885	Metabolic
ATPsynO	FBgn0016691	RRID: BDSC_43265	Metabolic
	FBgn0016691	RRID: BDSC_65180	Metabolic
CD98hc	FBgn0037533	RRID: BDSC_57746	Metabolic
CG5844	FBgn0038049	RRID: BDSC_35776	Metabolic
eIF3m/Tango7	FBgn0033902	RRID: BDSC_32879	Metabolic
eIF4E1	FBgn0015218	RRID: BDSC_34096	Metabolic
Hmgcr	FBgn0263782	RRID: BDSC_50652	Metabolic
Jheh3	FBgn0034406	RRID: BDSC_60021	Metabolic
ND-42	FBgn0019957	RRID: BDSC_32998	Metabolic
	FBgn0019957	RRID: BDSC_34526	Metabolic
ND-51	FBgn0031771	RRID: BDSC_36701	Metabolic
Sam-S	FBgn0005278	RRID: BDSC_36306	Metabolic

Baz	FBgn0000163	RRID: BDSC_35002	Cell polarity
	FBgn0000163	RRID: BDSC_39072	Cell polarity
Crb	FBgn0259685	RRID: BDSC_40869	Cell polarity
Sdt	FBgn0261873	RRID: BDSC_38988	Cell polarity

Prosalpha3	FBgn0261394	RRID: BDSC_77145	Proteosome
Prosalpha7	FBgn0023175	RRID: BDSC_33660	Proteosome
Prosbeta4	FBgn0032596	RRID: BDSC_32390	Proteosome
Prosbeta5	FBgn0029134	RRID: BDSC_34810	Proteosome

AGO1	FBgn0262739	RRID: BDSC_53293	RNA binding
CG10333	FBgn0032690	RRID: BDSC_34857	RNA binding
CG6015	FBgn0038927	RRID: BDSC_34565	RNA binding
Crn	FBgn0000377	RRID: BDSC_77146	RNA binding
eIF4A	FBgn0001942	RRID: BDSC_33970	RNA binding
mRNA-cap	FBgn0030556	RRID: BDSC_57297	RNA binding
pAbp	FBgn0265297	RRID: BDSC_53247	RNA binding

Ance	FBgn0012037	RRID: BDSC_36749	Signaling
CCKLR-17D1	FBgn0259231	RRID: BDSC_67865	Signaling
Ekar	FBgn0039916	RRID: BDSC_41852	Signaling
Mts	FBgn0004177	RRID: BDSC_38337	Signaling

RpL14	FBgn0017579	RRID: BDSC_80419	Translation
RpL22	FBgn0015288	RRID: BDSC_34828	Translation
RpL30	FBgn0086710	RRID: BDSC_80379	Translation
RpL32	FBgn0002626	RRID: BDSC_51746	Translation
RpL35A	FBgn0037328	RRID: BDSC_64935	Translation
RpL36	FBgn0002579	RRID: BDSC_53302	Translation
RpL39	FBgn0023170	RRID: BDSC_67901	Translation
RpL6	FBgn0039857	RRID: BDSC_34004	Translation
RpL7	FBgn0005593	RRID: BDSC_34600	Translation
RpS19a	FBgn0010412	RRID: BDSC_42774	Translation

CG11262	FBgn0036329	RRID: BDSC_67377	Transport/secretion
gammaCOP	FBgn0028968	RRID: BDSC_28889	Transport/secretion
inaD	FBgn0001263	RRID: BDSC_52313	Transport/secretion
Sar1	FBgn0038947	RRID: BDSC_32364	Transport/secretion
Sec61alpha	FBgn0086357	RRID: BDSC_35730	Transport/secretion

#### Metabolism

**Fig. 6.**
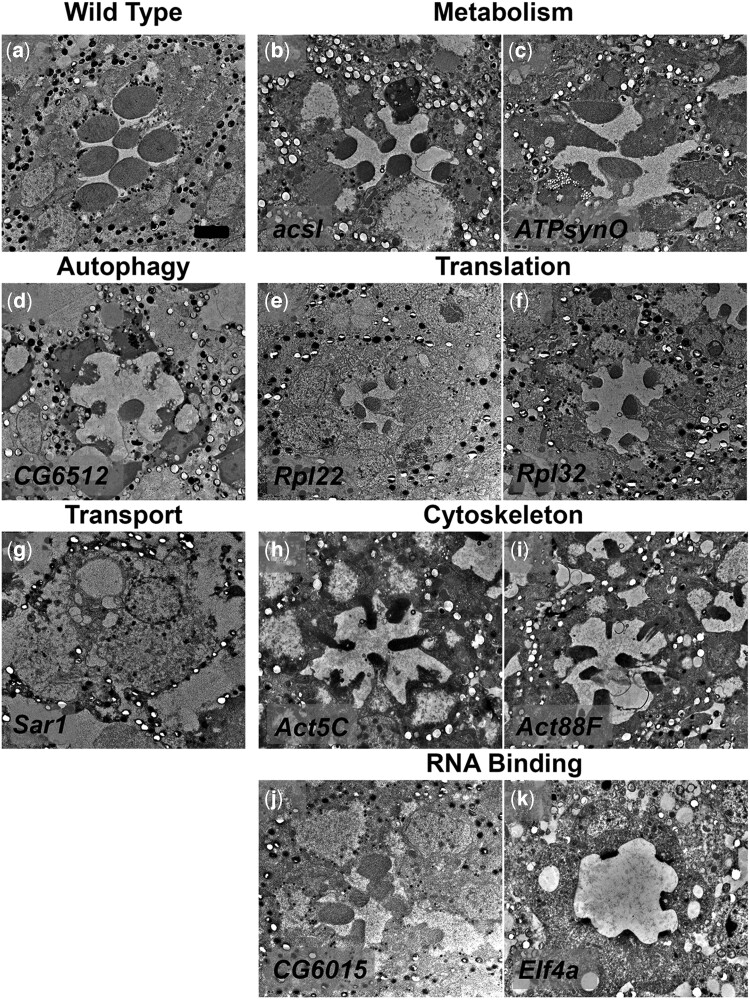
Transmission electron microscopy images of RNAi phenotypes of genes from diverse functional groups. Genes were placed into functional groupings using gene lists available from GLAD and any remaining genes were placed into the appropriate groups using associated GO terms, and previously reported functional data. Photoreceptor and rhabdomere structure in a single ommatidium of wildtype (Pph13-GAL4) (a), RNAi *acsl* (b), RNAi *ATPsynO* (c), RNAi *CG6512* (d), RNAi *Rpl22* (e), RNAi *Rpl32* (f), RNAi *Sar1* (g), RNAi *Act5C* (h), RNAi *Act88F* (i), RNAi *CG6015* (j), and RNAi *Elf4a* (k). Scale bar 2 μm.

This group encompasses genes with very diverse roles, and as such, diverse phenotypes would be expected. Acsl is involved in fatty acid synthesis. TEM analysis of Acsl knockdown flies shows a reduced rhabdomere phenotype, and the presence of some cells that may be degenerating based on increased electron-dense material (Fig. 6b). The phenotype observed is similar to photoreceptors degenerating as a result of *norpA* and *rdgB* mutations (Hsu *et al.* 2004). ATPsynO is a subunit of ATP synthase. This causes a more severe phenotype (Fig. 6c). It has been previously reported that knockdown of any of multiple subunits of ATP synthase resulted in an eye phenotype (Pletcher *et al.* 2019), but here we demonstrate the phenotype specific to photoreceptors.

#### Autophagy

TEM images of *CG6512* showed what appears to be a degenerative phenotype (Fig. 6d). This is in agreement with previous reports that autophagy machinery is needed for rhodopsin degradation, and photoreceptors with genetically depleted autophagy function degenerate (Wang *et al.* 2009; Midorikawa *et al.* 2010). CG6512 is homologous to the human mitochondrial peptidase AFG3L2. Mutations within AFG3L2 cause spinocerebellar ataxia and optic atrophy (Brussino *et al.* 1993; Charif *et al.* 2015).

#### Translation

Knockdown of multiple ribosomal genes resulted in reduced rhabdomeres and cell morphology like those observed in *Rpl22*, and *Rpl32* eyes (Fig. 6, e and f). This is likely due to reduced protein production capacity.

#### Transport/secretion

Genes in this group result in a severe phenotype. In almost all cases no detectable photoreceptors are identified as observed in *Sar1* (Secretion-associated Ras-related 1) knockdown photoreceptors (Fig. 6g). This result also highlights the temporal and spatial specificity of Pph13-GAL4. RNAi knockdown of *Sar1* utilizing Pph13-GAL4 did not result in the loss of pigment or cone cells nor was there any detectable rough eye phenotype associated with the knockdown of *Sar1*.

#### Cytoskeleton

RNAi lines targeting 2 different actin genes, *Act5C* and Act88F, resulted in the loss of a deep pseudopupil. TEM images show only rudimentary, poorly formed rhabdomeres (Fig. 6, h and i). This phenotype was expected as Actin is the key structural molecule of the microvilli which form rhabdomeres. However, the contribution of 2 different Actin genes was not predicted. Nonetheless, the hairpin target sequence of line 60347 (RRID: BDSC 60347) targeting *Act88F* also has a perfect 21 bp match with an exon in Act79B, and a 17 bp match with Act5C and could therefore knockdown 3 different actin genes in *Drosophila*.

#### RNA binding

Members of this group contribute to diverse roles in small RNA regulation and mRNA processing. CG6015 is the human *cdc40* ortholog (Flybase) and resulted in disrupted rhabdomere formation, and rhabdomere fusions (Fig. 6j). This phenotype is similar to that reported in mutants for the transmembrane protein Prominin (Zelhof *et al.* 2006)*. eIf4A* knockdown resulted in severely reduced rhabdomeres (Fig. 6k). Elf4A is a translation initiation factor and RNA helicase, and the phenotype is similar, albeit more severe, than that observed in Rpl22 or Rpl32 knockdown flies suggesting that this phenotype is also due to a reduced capacity for effective protein production.

### Concluding remarks

Through this work, we identified 64 genes involved in photoreceptor terminal differentiation, many of which have not been previously characterized in this context. Our approach was to target two sets of genes for RNAi knockdown using a photoreceptor-specific driver that initiates expression during differentiation. First, we targeted transcription factors to identify new genes that contribute to the regulation of terminal differentiation events, and second, we targeted genes involved in secretion and transport to identify factors involved in apical trafficking for rhabdomere morphogenesis. The diverse phenotypes that we observed suggest that we have identified genes involved in many aspects of terminal differentiation.

Interestingly, phenotypes associated with at least two of the hits from our screen were found to be due to off-target effects, *mamo* and CG11262. This is a well-documented challenge with RNAi screens (Ni *et al.* 2011). TRiP RNAi lines are reportedly quality controlled to filter out sequences for producing shRNAs that have more than 16 bp of complementarity to nontarget transcripts (Ni *et al.* 2011). However, we found that the target sequence of line 60111 (RRID: BDSC 60111) directed against *mamo* had at least 16 bp of complementarity to the mRNA sequences of 25 annotated genes, and this number expands to over 100 when the threshold is lowered to 15 bp, including multiple genes that are involved in establishing cell polarity. Thus the phenotype observed may be a combinatorial effect of knocking down several genes. With respect to CG11262, using RNAi line 67377 (RRID: BDSC 67377), we obtained an identical phenotype as observed with RNAi against *eys* and EYS protein is not detected. Subsequent analysis demonstrated the shRNA has 14 bp homology with the fifth coding exon of EYS. While it appears both these lines are outliers within the TRiP collection, it demonstrates the need for researchers to perform quality assessments of lines for hits obtained from RNAi screens and conducting appropriate follow-up experiments to confirm the results.

In future work, we will confirm and characterize the role of the genes that we have identified in this screen in photoreceptor differentiation, and we will further explore the new potential cellular processes and avenues this screen has revealed.

## Supplementary Material

jkac257_Supplementary_Figure_S1

jkac257_Supplementary_Figure_S2

jkac257_Supplementary_Figure_S3

jkac257_Supplementary_Figure_S4

jkac257_Supplementary_Figure_S5

jkac257_Supplementary_Figure_S6

jkac257_Supplemental_Figure_Legends

jkac257_Supplementary_Table_S1

jkac257_Supplementary_Table_S2

jkac257_Supplementary_Table_S3

jkac257_Supplementary_Table_S4

## Data Availability

All data necessary for confirming the conclusions in this paper are included in this article and supplemental tables. All reagents are readily available upon request or from public resource centers. The TRiP data generated in this study have been deposited to the RNAi Stock Validation and Phenotype (RSVP) database, which is publicly accessible through the DRSC/TRiP Functional Genomics Resources website. Supplemental material is available at G3 online.
