# Supplementary figures and images for: A TRiP RNAi screen to identify molecules necessary for Drosophila photoreceptor differentiation
Source: G3 (Bethesda). 2022 Oct 11;12(11):jkac257. doi: 10.1093/g3journal/jkac257 (PMC9635655; doi:10.1093/g3journal/jkac257)

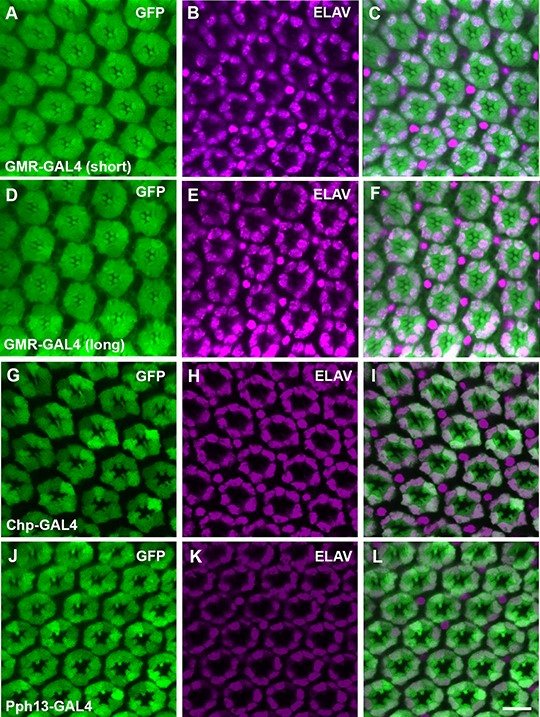

Supplement: jkac257_Supplementary_Figure_S1 [file jkac257_supplementary_figure_s1.jpeg]

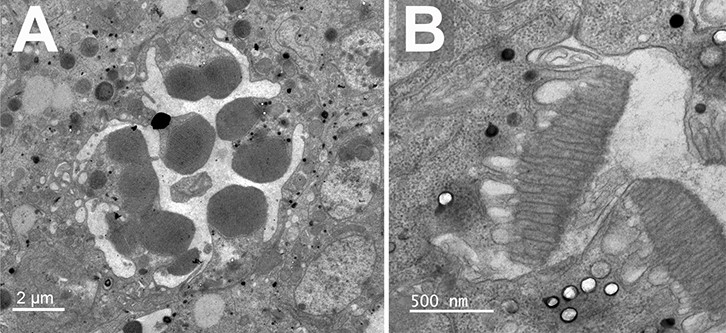

Supplement: jkac257_Supplementary_Figure_S2 [file jkac257_supplementary_figure_s2.jpeg]

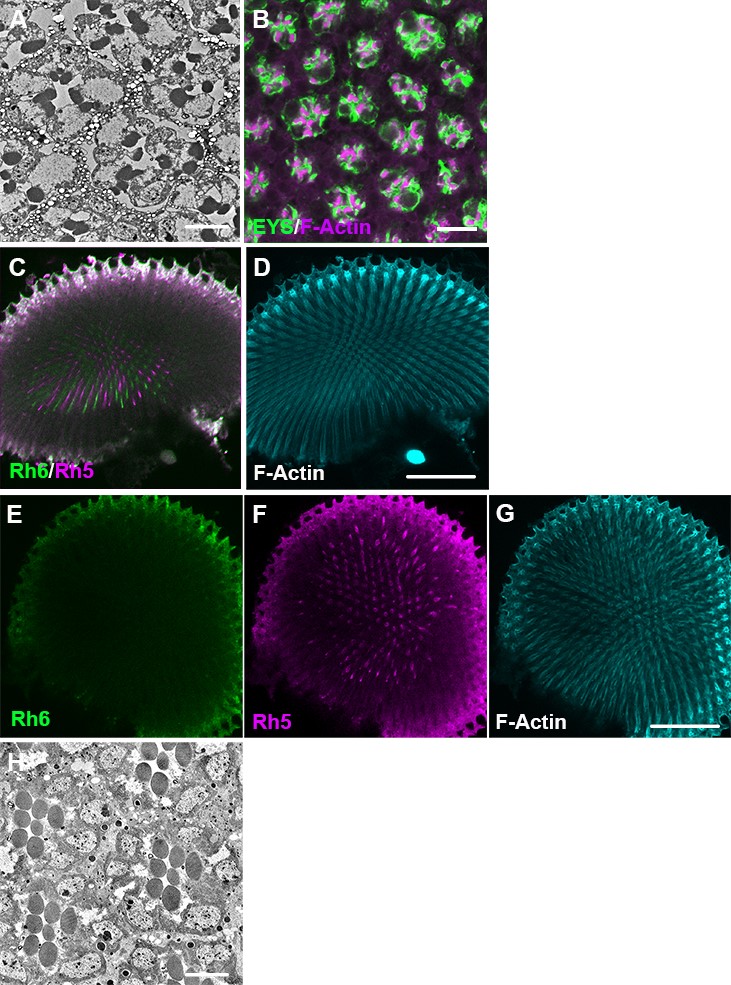

Supplement: jkac257_Supplementary_Figure_S3 [file jkac257_supplementary_figure_s3.jpeg]

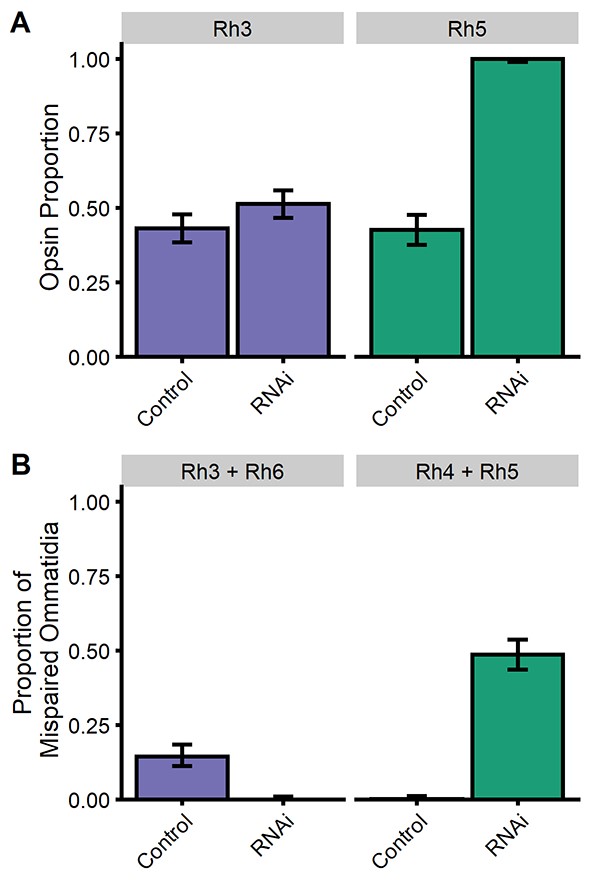

Supplement: jkac257_Supplementary_Figure_S4 [file jkac257_supplementary_figure_s4.jpeg]

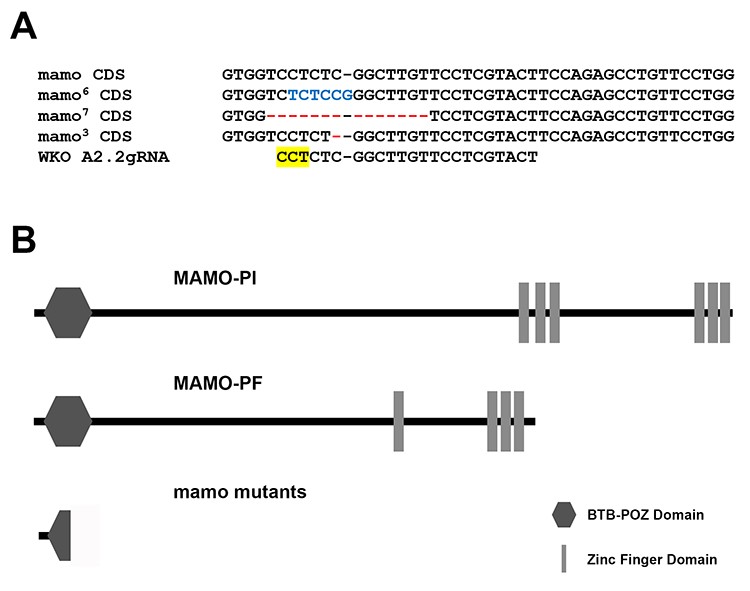

Supplement: jkac257_Supplementary_Figure_S5 [file jkac257_supplementary_figure_s5.jpeg]

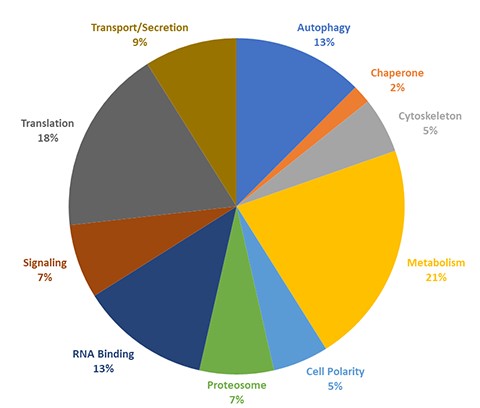

Supplement: jkac257_Supplementary_Figure_S6 [file jkac257_supplementary_figure_s6.jpeg]
