# Supplementary material for: A TRiP RNAi screen to identify molecules necessary for Drosophila photoreceptor differentiation
Source: G3 (Bethesda). 2022 Oct 11;12(11):jkac257. doi: 10.1093/g3journal/jkac257 (PMC9635655; doi:10.1093/g3journal/jkac257)
Supplement: jkac257_Supplemental_Figure_Legends [file jkac257_supplemental_figure_legends.docx]

**Figure S1: GFP reporter expression of retinal specific GAL4 lines in the developing retina at 72 hrs APF.** (A-C) GMR-GAL4 (short) driving GFP expression, green, countered stained with Elav, magenta. (D-F) GMR-GAL4 (long) driving GFP expression, green, counterstained with Elav, magenta. (G-I) Chp-GAL4 driving GFP expression, green, counterstained with Elav, magenta). (K-L) Pph13-GAL4 driving GFP expression, green, counterstained with Elav, magenta. Scale bar 10μm

**Figure S2: Transmission electron microscopy images of EcR DN and knirps RNAi knockdown.** (A) Photoreceptor and rhabdomere structure in a single ommatidium in which a dominant negative form of the Ecdysone Receptor (EcR DN) is expressed in photoreceptors via Pph13-GAL4. Scale bar is 2μm. (B) High magnification of the defective rhabdomere terminal web in a *knirps* RNAi knockdown photoreceptor. Scale bar is 500 nm.

**Figure S3: Phenotypes associated with RNAi line 60111, putative *mamo* knockdown, and *mamo* mutant photoreceptors.** (A) Transmission electron micrograph of phenotypes associated with RNAi line 60111, putative knockdown of *mamo*. Note the fused rhabdomeres, misshapen and misplaced rhabdomeres. Scale bar 5 μm (B) Immunofluorescence staining of putative RNAi knockdown of *mamo* adult retina with EYS (green) and phalloidin to mark the F-actin of the rhabdomeres (magenta). Scale bar 10 μm (C-D) Immunofluorescence staining of Rh6 (green) and Rh5 (magenta) opsin and F-actin in a wild-type retina. Scale Bar 75 um. (E-G) Immunofluorescence staining of Rh6 (green) and Rh5 (magenta) opsin and F-actin in RNAi knockdown of *mamo* in an adult retina. Scale bare 75 μm. (H) Transmission electron micrograph through a *mamo*^7^ mutant clone. Scale bar 5 μm.

**Figure S4: Quantification of Rh5 and Rh6 expression.** Dissociated ommatidia from control (generated by crossing *Pph13-GAL4* to *w^1118^*) or *mamo* RNAi adult retinas were manually scored based on opsin immunostaining patterns. The proportion of ommatidia expressing the indicated opsin or opsin combinations was calculated relative to the total number of ommatidia counted. (A) Shows the proportion of total ommatidia expressing Rh3 vs. Rh4 in R7 cells (purple) or Rh5 vs. Rh6 in R8 cells (green)**.** (B) shows the proportion of ommatidia exhibiting “mispaired” R7 and R8 photoreceptors, i.e. Rh3 paired with Rh6 (purple) as opposed to the normal Rh3/Rh5 pairing or Rh4 paired with Rh5 instead of with Rh6. Error bars represent 95% confidence intervals.

**Figure S5: CRISPR/Cas9 editing and generating of *mamo* mutant alleles.** (A) Schematic of induced CRISPR/Cas9 changes to *mamo* locus as compared to wildtype and location of gRNA WKO A2.2gRNA. The yellow highlighted region represents the PAM sequence. All three mutants lead to an early termination of the coding region. (B) Schematic of full-length Mamo protein isoforms as compared to the three Mamo mutants generated.

**Figure S6: Functional groups of positive RNAi hits.** The 57 genes were placed into broad categories based upon GLAD and GO terms.
