# Supplementary material for: A TRiP RNAi screen to identify molecules necessary for Drosophila photoreceptor differentiation
Source: G3 (Bethesda). 2022 Oct 11;12(11):jkac257. doi: 10.1093/g3journal/jkac257 (PMC9635655; doi:10.1093/g3journal/jkac257)
Supplement: jkac257_Supplementary_Table_S3 [file jkac257_supplementary_table_s3.docx]

| **Table S3: Primers for sequencing across *mamo* CRISPR sites** | |  |
| --- | --- | --- |
|  |  |  |
| **Primer** | **Sequence** |  |
| mamo A2.1 For | 5’-GTCATTCCCTGCTGCATTCCATAC-3’ |  |
| mamo A2.1 Rev | 5’-TCGTACCTATAATCTCGGACATCTTAT-3’ |  |
| mamo A2.1 seq | 5’-GTGACGTCCACCAGGCTCC-3’ |  |
|  |  |  |
| mamo A2.2 For | 5’-CACTCGCATGCTCTGGTATTTG-3’ |  |
| mamo A2.2 Rev | 5’-CTGATCTGAACAAAAGGACCACCC-3’ |  |
| mamo A2.2 seq | 5’-CACCTCGCCCTTGTACATGAAT-3’ |  |
